# Supplementary material for: Learning curve and surgical outcome of robotic assisted colorectal surgery with ERAS program
Source: Sci Rep. 2022 Nov 29;12:20566. doi: 10.1038/s41598-022-24665-w (PMC9709162; doi:10.1038/s41598-022-24665-w)
Supplement: Supplementary file 1 — Supplementary Information 1. [file 41598_2022_24665_MOESM1_ESM.zip › LSC Raw data-Ñ[▒K/056/OP_ñw╝╨░O▒Kñσ.pdf]

正式摘要(電子簽章：00000181824179)

請選擇...reporter

臺中榮民總醫院

Operation Note

[redacted]

- 手術日期：2019/12/12
- 手術醫師：蔣鋒帆，CR 林昌霖，CR 江世偉，R 陳瑋夫
- Anesthesia : GE
- Position : Lithotomy
- Aseptic agents : Betadine Alcohol , Betadine Aqua

Diagnosis

- Pre-operative diagnosis : Malignant tumor of rectum, FOR LAPA-OP
- Post-operative diagnosis : Rectal cancer, s/p CCRT, s/p Laparscopic Total mesocolon excision on 20191212, ycT3N0M0.

Surgical Indication

- For curative resection

Operation

- 1. Laparoscopic assisted low anterior resection with end-to-end anastomosis, EndoGIA 60/4.1mm*2, CDH 33mm, re-inforcement of silk (-), air leak test : no leakage.
  2. High ligation IMA below left colic artery.
  3. Cut margin: proximal 15 cm, distal 1.5 cm.
  4. Take down of splenic flexure: (-)
  5. A Fr.12 Penrose drain placed in the pelvis.
  6. Loop ileostomy on RLQ.

Finding

- 1. One 0.5x0.5cm Borrmann type II tumor at lowe rectum, which invaded through the muscular propria and into pericolorectal tissue. no enlarged lymph nodes at mesorectum were noted, ycT3N0M0.
  2. There was no palpable liver metastasis, no visible peritoneal seeding.

Procedure

- General endotracheal anesthesia was induced and then the patient was placed in modified Trendelenburg lithotomy position. Preoperative antibiotics were given. A Foley catheter and nasogastric tube were placed. The abdomen was prepped and draped in standard sterile fashion. A small vertical incision was made above the umbilicus and a 12mm trochar was inserted into the peritoneal cavity by open method. Peumoperitoneum was established with CO2 inflation. Laparoscopy was inserted and then another 12mm trochars were inserted over RLQ. Three 5mm trochars were inserted over RUQ, LUQ and LLQ of abdomen, respectively. The operative findings were mentioned as above. The small bowel was inspected and retracted to the right by grasper. The sigmoid colon was then held by grasper and the peritoneum overlying the mesentery was then scored with electrocautery. The inferior mesentery artery was identified, ligated above the left colic artery with endoclips, and transected, so was inferior mesentery vein. Dissection was done above the Gerota's fascia and toward the peritoneal attachments. The colon was mobilized from retroperitoneum overlying the sacral promontory and into the pelvis. The incision was extended laterally and medially across the peritoneal reflection. The mesorectum was then mobilized using sharp dissection. The mesorectum was then mobilized using sharp dissection until the levator ani muscle was identified. An endoscopic stapler was then used to divide the rectum and then laparoscope was removed. A midline incision was made at LLQ region. The sigmoid colon was

- delivered into the incision. It was determined that a 33 mm circular stapler could be accommodated. End to end anastomosis was performed. A purse-string suture of 3-0 Prolene was placed, the anvil was inserted, and then the purse-string suture was tied. The anal canal was irrigated with distil water and then the circular stapler was introduced through the anus and pierced through the mid-portion of the stapler line. The proximal bowel with anvil in place was engaged with the circular stapler and the stapler was then fired. Two complete donuts were obtained and submitted to pathology. The anastomosis was tested for leaks with irrigation solution and sir introduced through the rectum and was found to be patent and intact. One 12 Fr. Penrose was placed at pelvis. The fascia of LLQ wound was closed with interrupted sutures of No.1 Vicryl. The fascia of the two 12 mm trochar wound was closed with 1-0 Dexon. The loop ileostomy was matured by 4-O monocryl at RLQ. Skin was closed by 3-O nylon. The patient tolerated the procedure well and was brought to the POR in stable condition.
- Estimated blood loss: minimum
- 器械數量: 正確
- 紗布數量: 正確

Image

- PAINT
  - colon & rectum

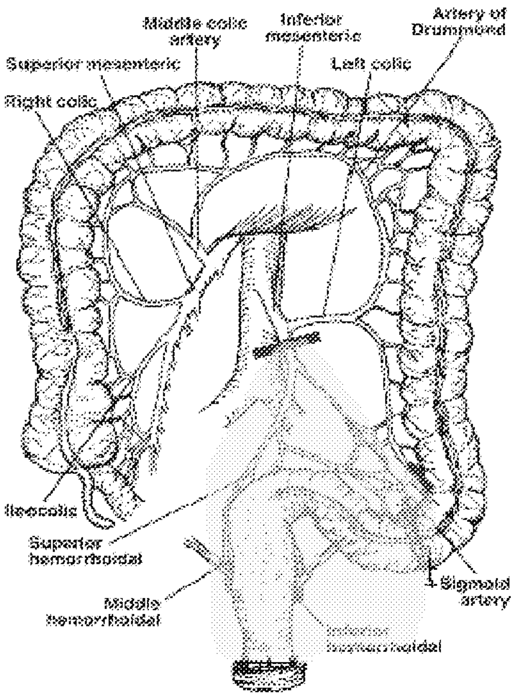

  - abdomen

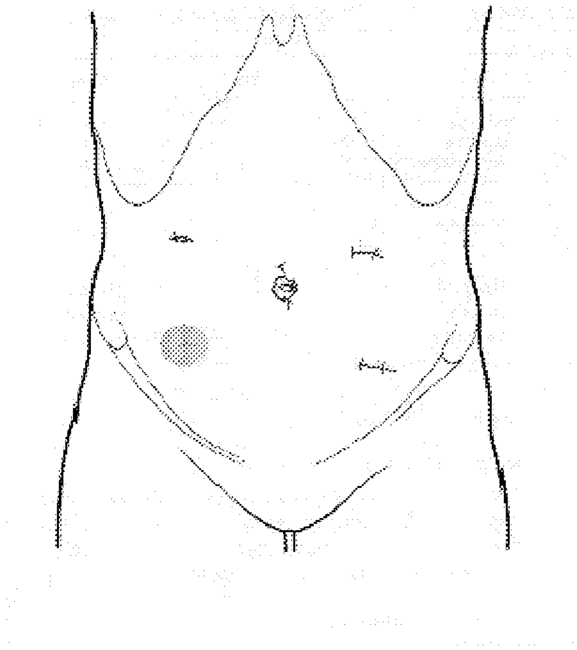

編輯主任 2062F蔣鋒帆、主治 2320B林昌霖

正式摘要(電子簽章：000000181824179)

請選擇...reporter

doctorRank:B
